# 4-Tosyl-1-oxa-4-aza­spiro­[4.5]deca-6,9-dien-8-one

**DOI:** 10.1107/S1600536809001524

**Published:** 2009-02-28

**Authors:** Ping Yin, Wen-Wen Zhang, Hai-Bin Yin, Ling He, Wen-Cai Huang

**Affiliations:** a Department of Medicinal Chemistry, West China School of Pharmacy, Sichuan University, Chengdu 610041, People’s Republic of China; b Guangzhou Yuantong Pharmaceutical Technology Co Ltd, Guangzhou 510610, People’s Republic of China; c Department of Pharmaceutical and Bioengineering, School of Chemical Engineering, Sichuan University, Chengdu 610065, People’s Republic of China

## Abstract

In the mol­ecule of the title compound, C_15_H_15_NO_4_S, the two six-membered rings are almost parallel to each other [dihedral angle = 1.87 (9)°] and perpendicular to the mean plane through the five-membered ring [dihedral angles of 89.98 (10) and 89.04 (10)°]. The crystal structure is stabilized by inter­molecular C—H⋯O hydrogen-bonding inter­actions.

## Related literature

For general background to the catalytic oxidation of phenol derivatives using transition metal complexes, see: Bernini *et al.* (2006 ▶); Cheung *et al.* (2005 ▶). For puckering parameters, see: Cremer & Pople (1975 ▶).
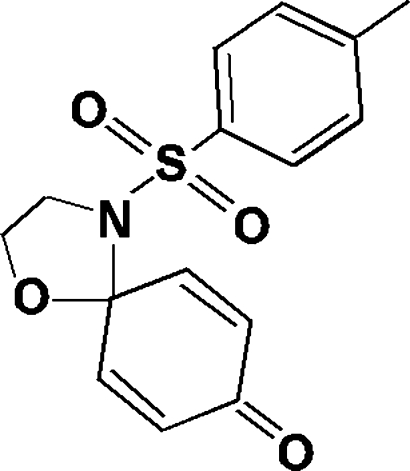


## Experimental

### 

#### Crystal data

C_15_H_15_NO_4_S
*M* *_r_* = 305.34
Monoclinic, 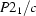

*a* = 11.882 (3) Å
*b* = 14.973 (6) Å
*c* = 8.369 (5) Å
β = 107.00 (3)°
*V* = 1423.9 (11) Å^3^
*Z* = 4
Mo *K*α radiation
μ = 0.24 mm^−1^
*T* = 294 K
0.30 × 0.25 × 0.20 mm

#### Data collection

Enraf–Nonius CAD-4 diffractometer
Absorption correction: none
2996 measured reflections
2625 independent reflections
1526 reflections with *I* > 2σ(*I*)
*R*_int_ = 0.008
3 standard reflections every 300 reflections intensity decay: 1.6%

#### Refinement

*R*[*F* ^2^ > 2σ(*F* ^2^)] = 0.046
*wR*(*F* ^2^) = 0.119
*S* = 1.02
2625 reflections
192 parameters
H-atom parameters constrained
Δρ_max_ = 0.30 e Å^−3^
Δρ_min_ = −0.27 e Å^−3^

### 

Data collection: *DIFRAC* (Gabe & White, 1993 ▶); cell refinement: *DIFRAC*; data reduction: *NRCVAX* (Gabe *et al.*, 1989 ▶); program(s) used to solve structure: *SHELXS97* (Sheldrick, 2008 ▶); program(s) used to refine structure: *SHELXL97* (Sheldrick, 2008 ▶); molecular graphics: *ORTEP-3 for Windows* (Farrugia, 1997 ▶); software used to prepare material for publication: *SHELXL97*.

## Supplementary Material

Crystal structure: contains datablocks global, I. DOI: 10.1107/S1600536809001524/rz2288sup1.cif

Structure factors: contains datablocks I. DOI: 10.1107/S1600536809001524/rz2288Isup2.hkl

Additional supplementary materials:  crystallographic information; 3D view; checkCIF report

## Figures and Tables

**Table 1:** Hydrogen-bond geometry (Å, °)

*D*—H⋯*A*	*D*—H	H⋯*A*	*D*⋯*A*	*D*—H⋯*A*
C2—H2⋯O4^i^	0.93	2.48	3.211 (4)	134
C15—H15*B*⋯O2^ii^	0.96	2.57	3.520 (5)	171

Symmetry codes: (i) 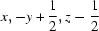; (ii) 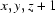.

**Fig. 1.**
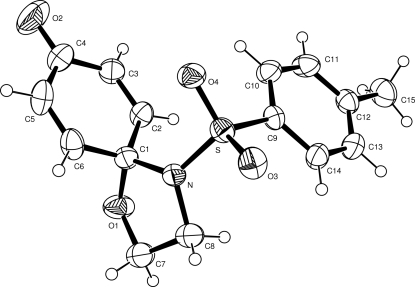
The molecular structure of the title compound, with displacement ellipsoids drawn at the 50% probability level.

**Fig. 2.**
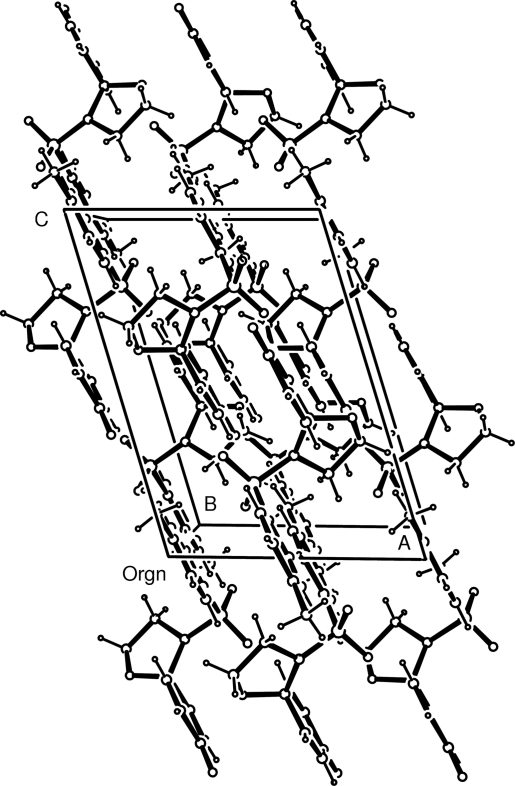
Crystal packing of the title compound approximately viewed along the b axis.

## supplementary crystallographic information

### Comment

In the course of our studies aimed to prepare a substituted quinone from the corresponding aromatic ether by catalytic oxidation using transition metal complexes (Cheung *et al.*, 2005; Bernini *et al.*,2006), the title compound was unexpectedly obtained in about 70% yield. In the molecule of the title compound (Fig. 1) the C1–C6 and C9–C14 six-membered rings are almost parallel to each other(dihedral angle 1.87 (9)°) and perpendicular to the mean plane through the O1/N1/C1/C7/C8 ring, forming dihedral angles of 89.98 (10) and 89.04 (10)°, respectively. The five-membered ring adopts an envelope conformation, with puckering parameters *Q*2 = 0.269 (3) Å and φ2 =104.4 (6)° ((Cremer & Pople, 1975). The crystal structure (Fig. 2) is enforced by intermolecular C—H···O hydrogen bonds (Table 1).

### Experimental

A solution of 2-phenoxyethyl-*p*-toluenesulfon amide (1 mmol) and indobenzene diacetate (1.5 mmol) in dichloromethane was charged in a reaction flask and 4 A molecular sieves was added. Then, the mixture was stirred at 303 K under a nitrogen atmosphere for 4 h. After cooling to room temperature, the resulting mixture was filtered and the solvent was removed under vacuo. The residue was purified by flash column chromatography on silica gel with petroleum ether/ethyl acetate (5:1 *v*/*v*) as the eluent. Colorless crystals suitable for X-ray analysis were obtained by slow evaporation in a cyclohexane/ether solution (5:1 *v*/*v*) at room temperature.

### Refinement

H atoms were positioned geometrically (C—H = 0.93–0.98 Å) and refined using a riding model, with *U*_iso_(H) = 1.2–1.5*U*_eq_(C).

### Crystal data

**Table:**

C_15_H_15_NO_4_S	*F*(000) = 640
*M**_r_* = 305.34	*D*_x_ = 1.424 Mg m^−^^3^
Monoclinic, *P*2_1_/*c*	Mo *K*α radiation, λ = 0.71073 Å
*a* = 11.882 (3) Å	Cell parameters from 22 reflections
*b* = 14.973 (6) Å	θ = 4.5–7.7°
*c* = 8.369 (5) Å	µ = 0.24 mm^−^^1^
β = 107.00 (3)°	*T* = 294 K
*V* = 1423.9 (11) Å^3^	Block, colourless
*Z* = 4	0.30 × 0.25 × 0.20 mm

### Data collection

**Table:**

Enraf–Nonius CAD-4 diffractometer	*R*_int_ = 0.008
Radiation source: fine-focus sealed tube	θ_max_ = 25.5°, θ_min_ = 1.8°
graphite	*h* = −14→3
ω/2–θ scans	*k* = −18→0
2996 measured reflections	*l* = −9→10
2625 independent reflections	3 standard reflections every 300 reflections
1526 reflections with *I* > 2σ(*I*)	intensity decay: 1.6%

### Refinement

**Table:**

Refinement on *F*^2^	Primary atom site location: structure-invariant direct methods
Least-squares matrix: full	Secondary atom site location: difference Fourier map
*R*[*F*^2^ > 2σ(*F*^2^)] = 0.046	Hydrogen site location: inferred from neighbouring sites
*wR*(*F*^2^) = 0.119	H-atom parameters constrained
*S* = 1.02	*w* = 1/[σ^2^(*F*_o_^2^) + (0.0523*P*)^2^ + 0.2087*P*] where *P* = (*F*_o_^2^ + 2*F*_c_^2^)/3
2625 reflections	(Δ/σ)_max_ < 0.001
192 parameters	Δρ_max_ = 0.30 e Å^−^^3^
0 restraints	Δρ_min_ = −0.27 e Å^−^^3^

### Special details

**Table:**

Geometry. All e.s.d.'s (except the e.s.d. in the dihedral angle between two l.s. planes) are estimated using the full covariance matrix. The cell e.s.d.'s are taken into account individually in the estimation of e.s.d.'s in distances, angles and torsion angles; correlations between e.s.d.'s in cell parameters are only used when they are defined by crystal symmetry. An approximate (isotropic) treatment of cell e.s.d.'s is used for estimating e.s.d.'s involving l.s. planes.
Refinement. Refinement of *F*^2^ against ALL reflections. The weighted *R*-factor *wR* and goodness of fit *S* are based on *F*^2^, conventional *R*-factors *R* are based on *F*, with *F* set to zero for negative *F*^2^. The threshold expression of *F*^2^ > σ(*F*^2^) is used only for calculating *R*-factors(gt) *etc*. and is not relevant to the choice of reflections for refinement. *R*-factors based on *F*^2^ are statistically about twice as large as those based on *F*, and *R*- factors based on ALL data will be even larger.

### Fractional atomic coordinates and isotropic or equivalent isotropic displacement parameters (Å^2^)

**Table:**

	*x*	*y*	*z*	*U*_iso_*/*U*_eq_	
S1	0.28106 (6)	0.58803 (5)	0.06825 (9)	0.0407 (2)	
O1	0.09259 (19)	0.57997 (15)	−0.3866 (3)	0.0638 (7)	
O2	−0.1639 (2)	0.7616 (2)	−0.1273 (4)	0.1088 (11)	
O3	0.20120 (17)	0.62375 (14)	0.1501 (3)	0.0500 (5)	
O4	0.35194 (17)	0.51210 (13)	0.1369 (3)	0.0531 (6)	
N1	0.20196 (18)	0.55681 (15)	−0.1165 (3)	0.0408 (6)	
C1	0.0935 (2)	0.60359 (18)	−0.2213 (4)	0.0420 (7)	
C2	0.0995 (3)	0.70272 (19)	−0.2139 (4)	0.0448 (7)	
H2	0.1650	0.7308	−0.2304	0.054*	
C3	0.0165 (3)	0.7524 (2)	−0.1852 (4)	0.0532 (8)	
H3	0.0239	0.8142	−0.1869	0.064*	
C4	−0.0868 (3)	0.7143 (3)	−0.1509 (5)	0.0648 (10)	
C5	−0.0943 (3)	0.6167 (2)	−0.1518 (4)	0.0612 (9)	
H5	−0.1587	0.5897	−0.1301	0.073*	
C6	−0.0124 (3)	0.5663 (2)	−0.1826 (4)	0.0523 (8)	
H6	−0.0207	0.5046	−0.1802	0.063*	
C7	0.1620 (3)	0.5036 (2)	−0.3849 (4)	0.0662 (10)	
H7A	0.1950	0.5048	−0.4780	0.079*	
H7B	0.1151	0.4498	−0.3926	0.079*	
C8	0.2563 (3)	0.5062 (2)	−0.2247 (4)	0.0613 (9)	
H8A	0.2779	0.4465	−0.1815	0.074*	
H8B	0.3257	0.5364	−0.2365	0.074*	
C9	0.3779 (2)	0.67304 (18)	0.0454 (3)	0.0383 (7)	
C10	0.3497 (3)	0.7621 (2)	0.0559 (4)	0.0493 (8)	
H10	0.2795	0.7780	0.0765	0.059*	
C11	0.4264 (3)	0.8267 (2)	0.0357 (4)	0.0536 (9)	
H11	0.4072	0.8865	0.0431	0.064*	
C12	0.5312 (3)	0.8055 (2)	0.0047 (4)	0.0472 (8)	
C13	0.5568 (3)	0.7168 (2)	−0.0071 (4)	0.0535 (8)	
H13	0.6266	0.7012	−0.0288	0.064*	
C14	0.4821 (2)	0.6503 (2)	0.0123 (4)	0.0487 (8)	
H14	0.5012	0.5907	0.0034	0.058*	
C15	0.6133 (3)	0.8785 (2)	−0.0157 (4)	0.0669 (10)	
H15A	0.5717	0.9187	−0.1021	0.100*	
H15B	0.6783	0.8526	−0.0456	0.100*	
H15C	0.6421	0.9105	0.0875	0.100*	

### Atomic displacement parameters (Å^2^)

**Table:**

	*U*^11^	*U*^22^	*U*^33^	*U*^12^	*U*^13^	*U*^23^
S1	0.0436 (4)	0.0356 (4)	0.0419 (4)	−0.0024 (4)	0.0108 (3)	0.0011 (4)
O1	0.0715 (15)	0.0670 (15)	0.0453 (13)	0.0231 (13)	0.0053 (11)	−0.0080 (12)
O2	0.0677 (17)	0.102 (2)	0.170 (3)	0.0206 (17)	0.0560 (19)	−0.020 (2)
O3	0.0541 (12)	0.0512 (12)	0.0523 (13)	−0.0066 (10)	0.0274 (11)	−0.0068 (10)
O4	0.0538 (13)	0.0428 (12)	0.0567 (13)	0.0034 (10)	0.0068 (10)	0.0121 (10)
N1	0.0385 (13)	0.0350 (13)	0.0467 (14)	0.0030 (10)	0.0090 (11)	−0.0056 (11)
C1	0.0415 (16)	0.0367 (17)	0.0463 (17)	0.0037 (13)	0.0106 (13)	−0.0020 (13)
C2	0.0433 (17)	0.0389 (17)	0.055 (2)	−0.0021 (14)	0.0191 (15)	0.0070 (15)
C3	0.0524 (19)	0.0370 (17)	0.069 (2)	0.0035 (15)	0.0153 (17)	−0.0028 (16)
C4	0.046 (2)	0.073 (3)	0.077 (3)	0.0122 (19)	0.0189 (18)	−0.006 (2)
C5	0.0384 (18)	0.068 (2)	0.076 (2)	−0.0077 (17)	0.0154 (17)	0.014 (2)
C6	0.0427 (17)	0.0369 (17)	0.070 (2)	−0.0053 (15)	0.0056 (16)	0.0093 (16)
C7	0.056 (2)	0.074 (3)	0.062 (2)	0.0135 (19)	0.0072 (18)	−0.0205 (19)
C8	0.0557 (19)	0.063 (2)	0.062 (2)	0.0097 (18)	0.0110 (17)	−0.0179 (18)
C9	0.0354 (16)	0.0394 (16)	0.0388 (16)	−0.0060 (13)	0.0088 (13)	0.0004 (13)
C10	0.0471 (18)	0.0431 (18)	0.065 (2)	−0.0050 (14)	0.0273 (16)	−0.0088 (16)
C11	0.064 (2)	0.0366 (17)	0.067 (2)	−0.0055 (16)	0.0306 (18)	−0.0081 (16)
C12	0.0453 (18)	0.050 (2)	0.0463 (19)	−0.0113 (15)	0.0135 (15)	−0.0031 (15)
C13	0.0389 (17)	0.060 (2)	0.064 (2)	−0.0015 (16)	0.0178 (16)	−0.0038 (18)
C14	0.0379 (16)	0.0429 (18)	0.063 (2)	0.0027 (14)	0.0107 (15)	−0.0012 (15)
C15	0.062 (2)	0.070 (2)	0.073 (2)	−0.0234 (19)	0.0262 (19)	−0.007 (2)

### Geometric parameters (Å, °)

**Table:**

S1—O3	1.427 (2)	C7—C8	1.478 (4)
S1—O4	1.431 (2)	C7—H7A	0.9700
S1—N1	1.626 (2)	C7—H7B	0.9700
S1—C9	1.763 (3)	C8—H8A	0.9700
O1—C7	1.409 (4)	C8—H8B	0.9700
O1—C1	1.424 (4)	C9—C10	1.384 (4)
O2—C4	1.218 (4)	C9—C14	1.388 (4)
N1—C8	1.469 (4)	C10—C11	1.372 (4)
N1—C1	1.503 (3)	C10—H10	0.9300
C1—C2	1.487 (4)	C11—C12	1.381 (4)
C1—C6	1.496 (4)	C11—H11	0.9300
C2—C3	1.313 (4)	C12—C13	1.373 (4)
C2—H2	0.9300	C12—C15	1.508 (4)
C3—C4	1.455 (4)	C13—C14	1.374 (4)
C3—H3	0.9300	C13—H13	0.9300
C4—C5	1.463 (5)	C14—H14	0.9300
C5—C6	1.314 (4)	C15—H15A	0.9600
C5—H5	0.9300	C15—H15B	0.9600
C6—H6	0.9300	C15—H15C	0.9600
			
O3—S1—O4	120.05 (13)	O1—C7—H7B	110.5
O3—S1—N1	106.48 (12)	C8—C7—H7B	110.5
O4—S1—N1	105.19 (12)	H7A—C7—H7B	108.7
O3—S1—C9	109.16 (13)	N1—C8—C7	102.7 (2)
O4—S1—C9	106.92 (13)	N1—C8—H8A	111.2
N1—S1—C9	108.57 (13)	C7—C8—H8A	111.2
C7—O1—C1	110.7 (2)	N1—C8—H8B	111.2
C8—N1—C1	109.7 (2)	C7—C8—H8B	111.2
C8—N1—S1	119.94 (18)	H8A—C8—H8B	109.1
C1—N1—S1	125.44 (18)	C10—C9—C14	119.7 (3)
O1—C1—C2	106.0 (2)	C10—C9—S1	120.7 (2)
O1—C1—C6	110.4 (2)	C14—C9—S1	119.5 (2)
C2—C1—C6	113.4 (2)	C11—C10—C9	119.3 (3)
O1—C1—N1	102.3 (2)	C11—C10—H10	120.4
C2—C1—N1	114.7 (2)	C9—C10—H10	120.4
C6—C1—N1	109.3 (2)	C10—C11—C12	121.9 (3)
C3—C2—C1	123.0 (3)	C10—C11—H11	119.0
C3—C2—H2	118.5	C12—C11—H11	119.0
C1—C2—H2	118.5	C13—C12—C11	117.8 (3)
C2—C3—C4	122.4 (3)	C13—C12—C15	121.9 (3)
C2—C3—H3	118.8	C11—C12—C15	120.2 (3)
C4—C3—H3	118.8	C12—C13—C14	121.9 (3)
O2—C4—C3	121.4 (4)	C12—C13—H13	119.1
O2—C4—C5	122.2 (3)	C14—C13—H13	119.1
C3—C4—C5	116.3 (3)	C13—C14—C9	119.4 (3)
C6—C5—C4	121.8 (3)	C13—C14—H14	120.3
C6—C5—H5	119.1	C9—C14—H14	120.3
C4—C5—H5	119.1	C12—C15—H15A	109.5
C5—C6—C1	123.0 (3)	C12—C15—H15B	109.5
C5—C6—H6	118.5	H15A—C15—H15B	109.5
C1—C6—H6	118.5	C12—C15—H15C	109.5
O1—C7—C8	105.9 (3)	H15A—C15—H15C	109.5
O1—C7—H7A	110.5	H15B—C15—H15C	109.5
C8—C7—H7A	110.5		

### Hydrogen-bond geometry (Å, °)

**Table:**

*D*—H···*A*	*D*—H	H···*A*	*D*···*A*	*D*—H···*A*
C2—H2···O4^i^	0.93	2.48	3.211 (4)	134
C15—H15B···O2^ii^	0.96	2.57	3.520 (5)	171

Symmetry codes: (i) *x*, −*y*+1/2, *z*−1/2; (ii) *x*, *y*, *z*+1.
